# 3DMesh-GAR: 3D Human Body Mesh-Based Method for Group Activity Recognition

**DOI:** 10.3390/s22041464

**Published:** 2022-02-14

**Authors:** Muhammad Saqlain, Donguk Kim, Junuk Cha, Changhwa Lee, Seongyeong Lee, Seungryul Baek

**Affiliations:** 1 AI Graduate School, Ulsan National Institute of Science and Technology, Ulsan 44919, Korea; saqlain@unist.ac.kr (M.S.); dukim@unist.ac.kr (D.K.); jucha@unist.ac.kr (J.C.); 2 Department of Computer Science and Engineering, Ulsan National Institute of Science and Technology, Ulsan 44919, Korea; changhwalee@unist.ac.kr (C.L.); skwithu@unist.ac.kr (S.L.)

**Keywords:** 3D human activity recognition, human body mesh estimation, feature extraction, deep learning, video understanding

## Abstract

Group activity recognition is a prime research topic in video understanding and has many practical applications, such as crowd behavior monitoring, video surveillance, etc. To understand the multi-person/group action, the model should not only identify the individual person’s action in the context but also describe their collective activity. A lot of previous works adopt skeleton-based approaches with graph convolutional networks for group activity recognition. However, these approaches are subject to limitation in scalability, robustness, and interoperability. In this paper, we propose 3DMesh-GAR, a novel approach to 3D human body Mesh-based Group Activity Recognition, which relies on a body center heatmap, camera map, and mesh parameter map instead of the complex and noisy 3D skeleton of each person of the input frames. We adopt a 3D mesh creation method, which is conceptually simple, single-stage, and bounding box free, and is able to handle highly occluded and multi-person scenes without any additional computational cost. We implement 3DMesh-GAR on a standard group activity dataset: the Collective Activity Dataset, and achieve state-of-the-art performance for group activity recognition.

## 1. Introduction

The purpose of human activity recognition is to identify what a human is doing in a scene using images and video frames or inertial, environmental, and physiological sensors data [1]. It is one of the most active areas of research and an immensely significant component of computer vision and computer graphics fields. Group activity recognition (GAR) is a subset of the human activity recognition problem that focuses on a group of people’s collective behavior resulting from their individual actions and interactions [2]. The GAR is a critical task in many domains for automatic analysis of human behavior, including intelligent surveillance, crowd monitoring, human-computer interaction, social behavior comprehension, robotics, sports video analysis, virtual reality, etc. [3]. Additionally, with the increasing population of elderly people, GAR is becoming a powerful tool to monitor functional, cognitive, and physical health at their homes or in hospitals [4]. Furthermore, it is critical to determine individual activities and interactions of people when recognizing group activity because these actions and interactions often constitute the group activity. Recent studies for this task have explored different methods for feature representations, such as optical flow [5], RGB (i.e., red, green, blue) image sequences [6,7], human skeletons [8], depth image sequences [9], and audio waves [10].

The majority of group activity recognition models either explicitly or implicitly examine human actions. Some studies identify individual activities and group activities in a combined framework using probabilistic graphical models [11] or neural networks that implement the capability of graphical models [12]. Other approaches simulate the relationship between individual persons’ activities and group activities by applying various pooling operations such as max-pooling [13] or attention pooling [14] on individual person representations. The temporal evolution of individual actions and group activities is another significant component in recognizing group activities. Recurrent Neural Networks (RNNs) have been used in several studies to recognize individual and group actions over time [14,15]. This strategy provides a concrete way to model group activities in video datasets. Moreover, some existing works attempt to inject temporal data via Convolutional Neural Networks (CNNs) on optical flow fields computed between two consecutive frames as an additional input to each time step of RNNs [15,16]. Recently, multi-stream convolutional networks beat RNNs in action identification tests [17], where CNN model temporal information on optical flow fields. However, this method can be expensive due to the computation of optical flow for numerous individuals and several forward runs of CNNs on the input optical flow field for all people in the video.

Researchers have proposed new action detection methods that can run on simpler hardware with few restrictive constraints in recent years. These methods do not need high-resolution cameras, special bodysuits, or in-studio recording but only require some scaled cameras for capturing the persons who are doing their everyday activities [18,19,20,21,22]. Moreover, various recent studies have used Deep Neural Networks (DNNs) to create 2D or 3D skeleton data with pose and shape information from a single RGB image, which are further used for action recognition tasks [3,8,23]. Unfortunately, most of the previous methods need more computation power and fail to get the required results for multi-person action recognition due to high occlusions, complicated backgrounds, a large variety of scenes and appearances, and depth uncertainties [24]. Additionally, current 3D skeleton methods show a deficiency in precise modeling of body-bone length distribution, which may predict impractical body structure such as abnormal limbs proportions and right-left asymmetry [25].

More recently, 3D human body mesh reconstruction aims to create full-body 3D meshes of humans in an image or video [26], and further, these meshes are being widely used for motion re-targeting [27], action recognition [28], virtual try-on [29], etc. A single-stage 3D mesh reconstruction method called ROMP (i.e., Regress all meshes in a One-stage fashion for Multiple 3D People) is presented for multi-person scenarios [21]. Therefore, this paper proposed a 3D human body mesh-based method for multi-person action recognition called 3DMesh-GAR. Our method is composed of three stages. Stage I contains a 3D mesh reconstruction network, which takes RGB image frames as the input, applies a mesh reconstruction network, and regresses the 3D body meshes of all people in the frame. Our mesh reconstruction network is computation cost-efficient, bounding box-free, and can learn pixel-level features in an end-to-end fashion. Stage II consists of the concatenation method and the feature extraction network. During the concatenation, all body meshes created in Stage I are merged into a single 3D body mesh by averaging them. Next, a feature extraction network is applied to the concatenated body mesh, converting the complex 3D mesh parameter into simple 2D trainable features. Finally, these features are treated as the input of a fully connected Deep Artificial Neural Network (D-ANN) in Stage III, which decides the action class of the input frame. We evaluate the performance of our method on a benchmark group activity recognition dataset called the Collective Activity Dataset [30]. The experimental results demonstrate that the proposed 3D mesh-based action recognition method obtained superior performance to the current state-of-the-art methods.

The rest of this paper is organized as follows: Section 2 contains related studies. Section 3 explains research materials and methods followed by this study. Dataset, experiments, and result analyses are provided in Section 4. The whole study is concluded in Section 5.

## 2. Related Work

We briefly review the recent literature on various deep learning-based and 3D skeleton-based group activity recognition approaches (Section 2.1). Then, we analyze recent one-stage/multi-stage methods designed for a single person and multi-person 3D human body mesh reconstruction (Section 2.2).

### 2.1. Group Activity Recognition Approaches

For the last decade, the computer vision research community has widely studied group activity recognition from video datasets. Most of these studies are based on visual features extracted using some 2D Convolutional Neural Networks (2D-CNNs) for each individual in an input frame and then building probability graphical networks on the prior knowledge for recognizing group activities [31,32,33,34]. Recently, CNNs models have achieved extensive success in activity recognition problems [14,15,35,36,37]. Additionally, ResNet [38], derivatives [39], and Inception [40] consolidate explicit knowledge flowing from initial to later feature extraction layers in the network via skip and summation connections. This strategy allows the training of deeper and more powerful models. With the advancements in deep learning techniques, feature extraction from input objects has been jointly optimized with the latest relational modeling methods such as Deep CNNs [41], Graph Neural Networks (GNNs) [42,43,44], Recurrent Neural Networks (RNNs) [38,45,46,47], and Transformers [48,49,50].

There have been several attempts to address the problem of group activity detection using probabilistic graphical models. Sun et al. [51] used a latent graph model for multi-target tracking, activity group localization, and group recognition. Hand-crafted features are used as input to the model in the initial probabilistic techniques. With the recent success of deep neural networks in various computer vision applications, these networks are now being used as feature extractors and inference engines in probabilistic group activity detection models. Deng et al. used CNNs as an initial classifier to come up with unary potentials [52]. They used a deep neural network to create the graphical model and performed messages traveling through the network to refine initial predictions. The authors presented multi-stream convolutional frameworks for group activity recognition in [2,53], in which new input modalities are simply included in the model by adding new convolutional streams. Li et al. proposed a real-time inference method for multi-person tracking and collective activity detection at individual, interaction, and group activity levels [54]. Simonyan and Zisserman [5] employed a two-stream CNNs architecture that can independently attain representation on optical flow assembled frames and RGB images. Azar et al. represented a CNNs based spatial relational method for group activity detection [41]. Wang et al. proposed an effective CNNs framework for action recognition from videos by dividing the input video into many chunks and applying a multi-stream method to combine each chunk with their corresponding part in a learnable way [17].

Some of the recent studies depend on spatio-temporal information extracted using RNNs. Ramanathan et al. proposed a multi-stage RNNs model to recognize only similar events in input videos by extinguishing irrelevant information [14]. Their model learns to recognize activities in videos while spontaneously attending to the main objects responsible for an activity. Ibrahim et al. [13] presented a deep architecture for modeling group activities in a principled structured temporal framework. The first part of their two-stage technique modeled individual-level activities, then merged all individual-level information to reflect collective activities. The Long Short-Term Memory (LSTM) network was used to represent the temporal representation of the model. Ibrahim et al. [37] introduced a hierarchical relational network that computed relational representations of persons based on graph structures that describe their potential relationships. Individual human representations and hypothetical relationship networks were provided to each related layer. Based on their connections with corresponding graphs, relational representations of each person were constructed. This method can be used to acquire relational feature representations that can successfully distinguish between different types of single-person and group activities. Another study looked into the use of RNNs for message passing [12]. They also proposed gating functions for learning the graph’s structure. A few studies also look at using structured RNNs to predict the scene context [15,55] or generate captions [16]. For group activity recognition, Li et al. suggested a two-stage semantic-based approach [16]. In the first stage, they implement the LSTM model to create captions for all video frames, while in the second stage, another LSTM model is implemented to identify the final activity class based on the created captions in the first stage.

Although attention mechanisms were originally designed for Natural Language Processing (NLP) problems, recently researchers have also proposed them for group activity detection by consolidating attention via pooling methods [56], graphs [57], or LSTM models [58]. Tang et al. joined attention mechanisms to get compact representations by allocating varying pooling weights to the various individual or group interactions [59]. Lu et al. proposed a spatio-temporal attention mechanisms-based method to utilize spatial configuration and temporal dynamic in a collective scene [60]. Moreover, attention-based models can also be applied for various modalities, such as motion [58] and pose [61]. Fernando et al. represented a temporal pooling function-based method for learning features of an input video through ranking machines. Later on, these features were used to recognize the input video with some classifier such as support vectors machines (SVMs) [62].

Graph Convolutional Networks (GCNs), a semi-supervised learning method, has recently become an emerging research topic in deep learning [63]. Some researchers have applied GCNs to recognize single-human activity [8,57] and group activity [42]. Wu et al. [42] proposed a flexible and efficient Actor Relation Graph (ARG) to simultaneously capture the appearance and position relation between actors. The connections in ARG were automatically learned using the GCNs from group activity videos in an end-to-end manner, and the inference on ARG were efficiently performed with standard matrix operations. Vaswani et al. proposed a transformer network that can learn long-term dependencies in a better way as compared to RNN due to its self-attention mechanism [45]. Girdhar et al. introduced a transformer network combined with 3D CNN representation for video action localization and action recognition [64]. Gavrilyuk et al. proposed an actor-transformer method for group activity recognition, which uses optical flow for temporal dynamics representation while pose information has been applied for interpreting spatial information of multiple people [65]. However, using optical flow information as input requires a high computational cost. Moreover, most of the current studies for group activity recognition consist of complex multi-stage architecture and required bounding boxes and well-designed hand-crafted features for each individual in the frame. Our method is simple, easy to use, bounding box free, and uses 3D mesh features for group activity recognition.

### 2.2. 3D Human Body Mesh Reconstruction Methods

Previous studies estimate monocular 3D-pose estimation for a single person in the form of a non-parametric 3D shape [66,67] or body skeleton [68,69,70,71,72]. The SMPL (i.e., Skinned Multi-Person Linear model) parametric model [73] has also been widely used for human body mesh recovery. The SMPL is adapted to convert a highly complicated 3D body mesh into a simple vector with very low dimensions, which can be regressed by an image [74]. Bogo et al. proposed the first optimization-based method called SMPLify, which can continuously train SMPL with the learned 2D joints [75].

Some recent studies applied deep neural networks in a multi-stage manner for direct SMPL parameters regression. These studies first approximate intermediary representations such as silhouettes and keypoints from the input images and then regress SMPL parameters by mapping them [76,77,78]. Some other studies regressed SMPL parameters directly from images, either by leveraging temporal learning [79,80] or complex model training methods [81,82]. Moreover, some researchers employed CNNs-based learning methods to get satisfactory results for 3D pose estimation [83,84,85]. Transfer learning methods have also been implemented to enhance 3D pose estimation using features obtained from 2D pose datasets [86,87,88]. Recently, Cha et al. proposed a self-supervised learning based method for 3D human body pose and shape estimation using only 2D images [89]. Their method does not require other type of supervision signals such as video-level, multi-view priors, or 2D/3D skeletons. However, all these methods achieved higher accuracy only for single-person problems, and it remains ambiguous how to use these methods for more common multi-person problems.

For multi-person 3D regression, most of the existing methods are composed of a multi-stage approach that provides the single-person model with a 2D person identifier to solve multi-person problems [90,91]. Recently, many researchers have been focusing on multi-person 3D pose estimation, for which they use a top-down paradigm [92,93]. In top-down methods, firstly, all individual person instances are detected, and then features are extracted using the bounding-box method, and finally, body joins locations are regressed using those features for each person [76,80,81,94]. Different multi-stage methods have also been proposed for this purpose using Faster Region-based Convolutional Neural Networks (R-CNN) [95], such as 3D Multi-Person Pose Estimation (3DMPPE) [96] and Localization Classification Regression Network (LCR-Net++) [93]. Moon et al. applied a prior person identification step and transferred all resized and cropped images of identified persons to the 3D pose estimation network [96]. However, top-down methods depend considerably on human detection for localization of each individual prior to body joint estimation within the identified bounding boxes [97,98]. These methods have no realization of persons who are out of the bounding boxes and their possible interaction. Moreover, human detection becomes unreliable for highly occluded scenarios, resulting in misleading the pose estimation of the targeted person with the nearby persons.

Recent bottom-up approaches for 3D mesh estimation do not require individual person detection and thus can achieve higher accuracy in the presence of multi-person scenarios [99,100]. These methods take all persons in a frame simultaneously and distinguish their joints in a better way. Fabbri et al. proposed a pose estimation method called Learning on Compressed Output (LoCO) for mapping the images into the volumetric heatmaps and using these heatmaps to estimate multi-person 3D poses through an encoder-decoder framework [101]. More recently, Zhang et al. represented a single-stage method that shows instances of multi-person in the space of spatial depth where all points are associated with their corresponding body meshes [26]. Their method can directly identify human body meshes through concurrently localizing human instance points and predicting corresponding 3D meshes. Sun et al. also proposed a single-stage method called ROMP for 3D mesh regression of multi-person scenarios [21]. ROMP can simultaneously identify mesh parameter map and body center heatmap, which are jointly used to predict a 3D person map on the pixel level. Their method achieved state-of-the-art results on the highly occluded and crowded dataset. So, we also used a single-stage 3D mesh reconstruction method as the backbone of our pipeline for 3D mesh regression from the group activity datasets.

## 3. Materials and Methods

**Figure 1:**
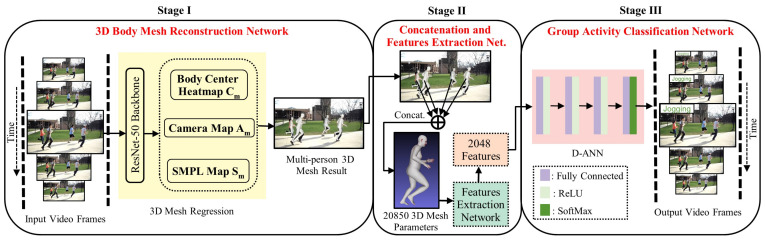
An overview of our 3DMesh-GAR framework for 3D body mesh-based group activity recognition. Stage I infers 3D body mesh reconstruction for each input RGB frame in a video using a 3D mesh regression model. Stage II provides the concatenation and features extraction networks used to concatenate all 3D body meshes of the frame into a single averaged 3D mesh and extract learnable features from the concatenated 3D body mesh, respectively. Finally, Stage III provides a fully-connected deep artificial neural network (D-ANN) trained with learnable mesh features to classify the group activities.

The details of our proposed method are given in this section. Figure 1 illustrates all the important elements of the proposed learning framework for group action recognition from 3D human body meshes. We first show how the 3D human body meshes are created in multi-person scenarios from simple RGB images (Section 3.1). Then, we introduce a concatenation method to aggregate all the meshes into a single 3D mesh and a feature extraction network to convert 3D mesh parameters into trainable features (Section 3.2). Finally, a fully connected deep neural network is presented to train and classify the group actions from the trainable features (Section 3.3).

### 3.1. 3D Body Mesh Reconstruction: Stage I

Stage I contains a 3D body mesh reconstruction network for body mesh regression from the multi-person dataset. As illustrated in our framework, a ResNet-50 [38] network is applied as the default backbone to the input RGB image of size 512×512 and extracts a backbone feature vector $yf\in R^{34\times H_{b}\times W_{b}}$ where $H_{b}$ and $W_{b}$ are the height and width of the backbone feature, respectively, their values are set to 128. From the backbone feature, three different head networks are built to find three types of maps, such as body center heatmap $\left(C_{m}\right)$ , camera map $\left(A_{m}\right)$ , and SMPL map $\left(S_{m}\right)$ . These maps comprehensively describe the estimated 3D body mesh information. The body center heatmap predicts the probability of all positions being people body centers. Using these position parameters, the camera map and SMPL maps are applied to get camera parameters and SMPL parameters, respectively, which are further gathered to define the 3D mesh parameter map $\left(P_{m}\right)$ . The size of all maps is given by n×H×W , where *n* represents the total number of channels, and *H* and *W* are the height and width of the maps, respectively. The value of both height and width of all the maps is set to 64. Each map is further elaborated in the following subsections.

#### 3.1.1. Body Center Heatmap: *C* ${}_{m}$ $\in R^{1\times H\times W}$

**Figure 2:**
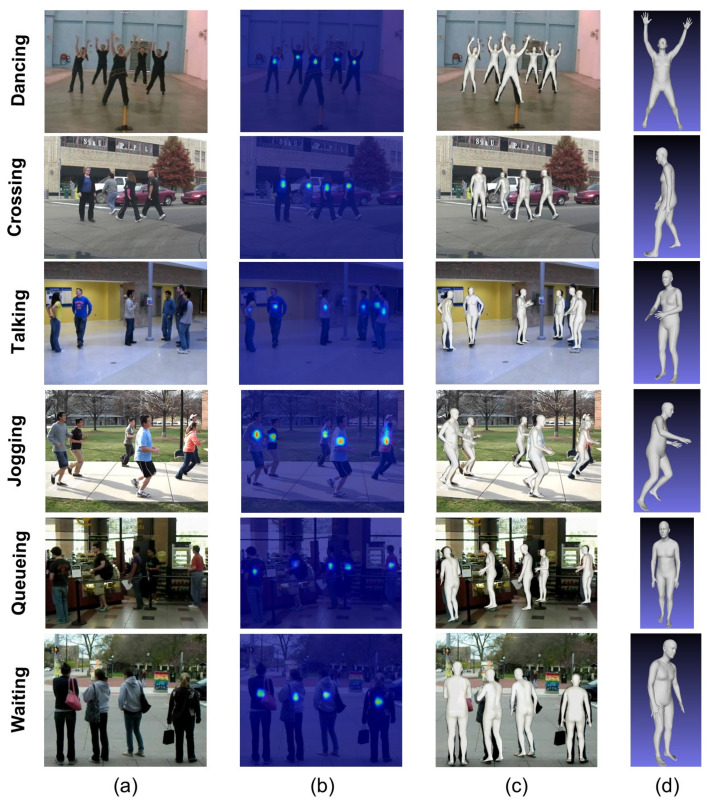
3D body mesh reconstruction examples: We applied a 3D mesh reconstruction network to create 3D body meshes for each individual person from the input RGB frames. Each row presents examples from the Collective Activity Dataset, and each column corresponds to (**a**) input RGB frames, (**b**) body center heatmaps of each person in the frame, (**c**) 3D body meshes of each person in the frame, and (**d**) concatenated 3D body mesh, respectively.

The $C_{m}$ map is the heatmap showing the 2D person body’s central point in the input RGB image. All body centers are presented as a Gaussian distribution in the $\left(C_{m}\right)$ , calculated by Gaussian kernel size *k* of all person centers in terms of their 2D bodies. The value of *k* is derived by the following equation:

(1) $$\begin{array}{c} k=k_{l}+{\left(\frac{d_{b-box}}{\sqrt{2}W}\right)}^{2}k_{r} \end{array}$$

where $k_{l}$ , $d_{b-box}$ , *W*, and $k_{r}$ are minimum kernel size, diagonal length of the human bounding box, the width of the body center heatmap, and variation range of *k*, respectively. The values of $k_{l}$ and $k_{r}$ were set to 2 and 5, respectively, by default. The body center heatmaps of all persons from different actions of the collective active dataset [30] are shown in Figure 2b.

#### 3.1.2. Camera Map: *A* ${}_{m}$ $\in R^{3\times H\times W}$

The camera map consists of three camera parameters, one 2D scale *s* parameter, and two translation $t=(t_{x},t_{y})$ parameters for each person in the image. The scale *s* represents the size and depth of the person’s body. Whereas the translation $t_{x}$ and $t_{y}$ , whose values range in (−1,1), represent a normalized translation of the person’s body relative to the center of the image on the *x*-axis and *y*-axis, respectively.

#### 3.1.3. SMPL Map: *S* ${}_{m}$ $\in R^{142\times H\times W}$

The $S_{m}$ map consists of 142-dimensional SMPL parameters, obtained by employing the SMPL parametric model for person body representation [73]. It allows the use of pose parameters θ and shape parameters β to describe the full 3D human body mesh. The pose parameters are defined as $\theta \in R^{6\times 22}$ , containing a 6D representation of 3D rotational information for 22 human body joints. The shape parameters are defined as $\beta \in R^{10}$ , which are parameterized by the top-10 principal components of the 3D shape space. An efficient mapping is established by applying an SMPL differentiable function that takes the θ and β parameters as input and results in a triangular body mesh $M\in R^{6890\times 3}$ with 6890 vertices. The 3D joints are reconstructed by a PM process, where *P* is defined as $P\in R^{K\times 6890}$ , which is an infrequent weight matrix that expresses the linear mapping through 6890 vertices of human body mesh *M* to the body joint *K*.

#### 3.1.4. Mesh Parameter Map: *P* ${}_{m}$ $\in R^{145\times H\times W}$

The mesh parameter map is a combination of the SMPL map and camera map. The 3D human body mesh parameters are estimated by considering the positions of SMPL and camera maps to the centers of the human bodies. A weakly-perspective camera model is implemented to estimate 3D human body joints $J=(x_{k},y_{k},z_{k})$ , where k=1…K . These 3D body joints are used to get 2D projection joints $\hat{J}=\left({\hat{x}}_{k},{\hat{y}}_{k}\right)$ , where ${\hat{x}}_{k}$ and ${\hat{y}}_{k}$ are derived by camera parameters as ${\hat{x}}_{k}=sx_{k}+t_{x}$ , ${\hat{y}}_{k}=sy_{k}+t_{y}$ , respectively. It helps the mesh reconstruction model to train with 2D pose datasets, which increases the generalization and strength.

#### 3.1.5. Collision-Aware Representation (CAR)

Conventionally, human body centers are defined using the center of bounding boxes. However, this method failed to identify the body centers in highly occluded scenes. Thus, Sun et al. [21] proposed a novel method called collision-aware representation (CAR) to define the human body centers in densely overlapping people cases. Using this method, a repulsion field is created, in which each body center is considered positively charged. These same charged body centers repel each other, and their radius of repulsion is the same as the size of the Gaussian kernel defined by Equation (1). So, the CAR plays a vital role in pushing apart the closer body centers in multi-person occluded cases and using these differential body centers to create a body center heatmap. The impact of CAR can be seen in the *Tracking* and *Jogging* rows of Figure 2, where heatmaps of occluded persons are successfully identified. Moreover, by sampling these body center heatmaps with mesh parameter maps, 3D mesh features are extracted for each individual person, as shown in Figure 2c.

### 3.2. Concatenation and Features Extraction: Stage II

After Stage I, we have a per-frame 3D body mesh for each individual. The Stage II of our framework contains two simple networks such as (1) concatenation network and (2) feature extraction network. The prior network gets the output of Stage I with multi-person 3D mesh results as its input and implements a concatenation function to find a single 3D body mesh by averaging all body meshes. The value of concatenated 3D body mesh $S^{3D}$ is derived as:

(2) $$\begin{array}{c} S^{3D}=\left(\frac{S_{1}^{3D}+S_{2}^{3D}+\cdots +S_{n}^{3D}}{n}\right),n=1\ldots N \end{array}$$

where *N* is the total number of persons in the input frame. As each input frame belongs to one of the group activities, all persons in that specific frame represent the corresponding activity. Thus, we concatenated all frames with different numbers of persons into a single 3D body mesh corresponding to a specific group activity class, see Figure 2d. The final 3D body mesh contains three types of parameters, such as 3D coordinates of body mesh, SMPL pose, and 2D coordinates of 2D keypoints of shapes (6890,3) , (72,1) , and (54,2) , respectively. So, the total number of raw parameters of a single 3D body mesh is 20,850 . The second network of Stage II takes the raw mesh parameters from the concatenation network as the input. It applies a simple non-trainable linear network to convert the input 3D mesh parameters into trainable parameters of size 2048.

### 3.3. Activity Classification Network: Stage III

After Stage II, we have trainable features for each input RGB image. Stage III contains a deep artificial neural network (D-ANN) that takes the trainable features as the input and passes them to the multiple hidden layers for training using activation functions. It gives the output with one of the group activity classes. A D-ANN is a multi-layer fully-connected neural network and comprises an input layer, several hidden layers, and an output layer. All nodes of one layer are connected to all other nodes in the next layer. Nowadays, D-ANN models are being used in several real-world applications because of their outstanding performances [102]. The success of deep learning models from the last decade is due to a combination of both theoretical progression such as improved learning rate methods, optimization techniques, availability of numerous big datasets, etc., and easy access to improved and cheap hardware resources such as multi-processor graphics cards or graphics processing units (GPUs) [103]. The D-ANN methods are now routinely implemented with impressive results in areas such as pattern recognition, image analysis, object detection, fault diagnosis, self-driving cars, speech recognition, natural language processing, and robotics, to name a few areas.

**Figure 3:**
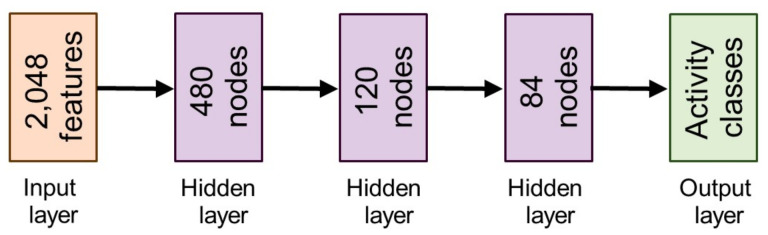
The architecture of a fully connected deep artificial neural network that forms Stage III of our pipeline.

We proposed a deep classification network by introducing a total of three hidden layers, each with Rectified Linear Units ( ReLU ) activation function. We preferred the ReLU activation function because it performs comparatively better than other sigmoidal activation functions in deep learning models and helps to get the best results on numerous benchmark problems in multiple domains [104]. Our fully connected deep neural network is illustrated in Figure 3, which contains an input layer with 2084 features from 3D human body mesh, 3 hidden layers with a different number of nodes such as 480, 120, and 84 nodes for the first, second, and third hidden layers, respectively, and an output layer with various possible activity classes. A simple learning function between nodes of different layers is calculated by an activation function, which can be defined as:

(3) $$\begin{array}{c} x=\Delta_{i}^{n}w_{i}\cdot x_{i}+b \end{array}$$

where $x_{i}=\left(x_{1},x_{2}\ldots .x_{n}\right)$ and $w_{i}=\left(w_{1},w_{2}\ldots w_{n}\right)$ are the values of the previous layer with a total of *n* nodes and their corresponding weights, and *b* is the bias value. The result of this learning function is passed through the non-linear ReLU activation function, which converts all negative values to zero while the remaining values are kept unchanged, as given in the following equation.

(4) $$\begin{array}{c} f(x)=max(0,x) \end{array}$$

The final action recognition is done at the output layer, whose nodes are equal to the number of input activity classes. The output layer uses the SoftMax activation function instead of ReLU , which gives the maximum probability value to the most precise activity class and vice versa.

## 4. Experiments and Result Analysis

In this section, we evaluate the effectiveness of the proposed method using a public benchmark group activity dataset, comparing our approach with current state-of-the-art methods for the same benchmark. A detailed explanation of the dataset is given in Section 4.1. The implementation details and hardware environment are explained in Section 4.2. The comparison of the proposed method with the state-of-the-art is described in Section 4.3. Result analysis and discussion are provided in Section 4.4. Limitations of this study and expected future works are given in Section 4.5.

### 4.1. Dataset

We conducted experiments on a widely-adopted public group activity dataset, namely the Collective Activity Dataset (CAD) [30]. It contains 44 short video sequences with 5 different group activities such as Crossing , Waiting , Queueing , Walking , and Talking . The group activity label for a specific frame is determined by an activity in which the majority of people are participating. Additionally, we used an augmented collective activity dataset with two additional activity classes such as Dancing and Jogging for further evaluation of our model. As this dataset is collected from outdoor activities with consumer hand-held digital cameras, the quality of some frames is not high. Thus, we pre-processed the dataset by keeping only those frames for which at least two body meshes are created and removing the other frames. We divide the whole dataset into 80% and 20% for the model’s training and validation.

To further analyze the performance of the proposed method for single-person as well as multi-person cases, we used NTU-60 RGB+D Dataset (NTU60) [105]. The original NTU60 dataset is very big and contains 56,880 videos samples with 60 different human action classes. We therefore used a subset of the original dataset with five random classes such as Back−Pain , Bow , Brush−Teeth , Cheer−Up , and Hands−Shaking . The first four classes contain single person actions and the last class contain multi-person action. We divided the dataset into 80% and 20% for the model’s training and validation.

### 4.2. Implementation Details

We used the ResNet-50 network as the backbone of the 3D mesh reconstruction network. All input RGB images are resized into 512×512 with zero padding and a similar aspect ratio. The size of backbone feature vectors resulting from ResNet-50 is 34×128×128 . These feature vectors are used to develop three head networks to find body center map, camera map, and SMPL map, each with output sizes of 1×64×64 , 3×64×64 , and 142×64×64 , respectively. The maximum number of persons in one frame whose 3D meshes can be created is manually set to 64. The values of the body center heatmap threshold and repulsion coefficient of CAR are set to 0.2 for each. Each person’s final 3D body mesh contains 20,850 mesh parameters that are converted to 2048 trainable features.

For the training of the D-ANN at Stage III, we use the ADAM optimizer [106] with fixed hyper-parameters $\beta_{1}=0.9$ , $\beta_{2}=0.999$ , and $\epsilon =10^{-10}$ . We adopt a mini-batch size of 1 sample with a learning rate ranging from 0.001 to 0.000001 . We train and validate the network for CAD and NTU60 datasets in 80 and 50 epochs, respectively. Our method is implemented on PyTorch based deep learning framework. The inference time for a single frame is approximately 1.6 ms on a single TITAN−RTX2080Ti GPU. The implementation code of our proposed method will be available online upon publication.

### 4.3. Comparison with the State-of-the-Art

We evaluate the proposed method on CAD dataset. The results are provided in Table 1, along with a comparison of different previous methods. Our 3D body mesh-based action recognition method outperforms all previous methods and shows the state-of-the-art results with 93.6% group activity recognition accuracy. All provided values have validation accuracy as pre-determined by the dataset authors. Meanwhile, our method slightly outperforms the recently published methods by Wu et al. [42] and Gavrilyuk et al. [65] with the validation accuracy of 90.0% and 92.8% , respectively. These outstanding results represent the effectiveness of the proposed method for group activity recognition using 3D body mesh reconstruction of multiple people scenarios.

To understand the effectiveness of our method, we compare the accuracy of each individual class of the CAD dataset with the previous state-of-the-art method [65] in Table 2. It can be seen that the proposed method outperformed the previous method to recognize the Crossing and Waiting classes with the accuracy of 88.8% and 96.8% , respectively. While, the accuracy of other classes such as Queueing (95.2%) , Talking (99.7%) , and Walking (86.7%) are also very high, their differences with [65] are negligible.

To further analyze the efficiency of the proposed method for single-person as well as multi-person cases, we compare the accuracy of a subset of the NTU60 dataset [105] with the previous state-of-the-art method proposed by Huang et al. [108] in Table 3. It is clear that our method outperformed the previous method with an overall validation accuracy of 92.2% . Our method also achieved higher accuracy for all individual classes such as Back−Pain (90.2%) , Bow (93.7%) , Brush−Teeth (88.1%) , and Cheer−Up (95.0%) , except one class Shaking−Hands (91.9%) . It shows that the proposed 3D body mesh-based action recognition method is the most suitable approach for general-purpose Guman Activity Recognition (GAR) in vast physical environments.

### 4.4. Results Analysis

**Figure 4:**
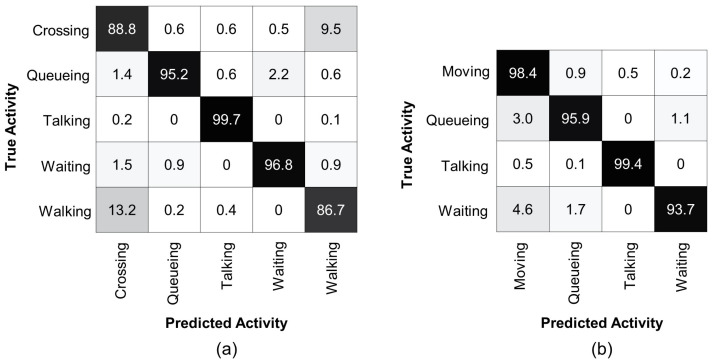
Confusion matrix for group activity recognition using Collective Activity Dataset. (**a**) denotes the confusion matrix for the original five classes. Most confusion occurs when distinguishing between the Crossing and Walking classes. (**b**) denotes the confusion matrix after merging the Crossing and Walking classes into a single Moving class. Our method achieves over 93% accuracy for each group activity class.

To analyze the performance of the proposed method, we present confusion matrices on the Collective Activity Dataset for multi-person activity recognition, see Figure 4. Similar to [65], our method also struggles to distinguish between the Crossing and Walking classes. The confusing ratio of the Crossing class with Walking is 9.5% , whereas, the Walking class with Crossing is 13.2% , as shown in Figure 4a. Therefore, following [15,50], we merged the Crossing and Walking classes into a single Moving class because there is no clear difference in physical appearance and pose of all persons of these classes and their 3D body mesh features are also similar. Figure 4b presents a confusion matrix of our method for group activity recognition with the merged Moving class. It is clear that our method achieves accuracy over 93% , with the least accuracy for the Waiting class (93.7%) . The accuracy for the Moving class, which is a combination of the Crossing and Walking classes, is also improved up to 98.4% . The most confusion occurs for recognizing the Queueuing class with Moving (3.0%) and the Waiting class with Moving (4.6%) .

**Figure 5:**
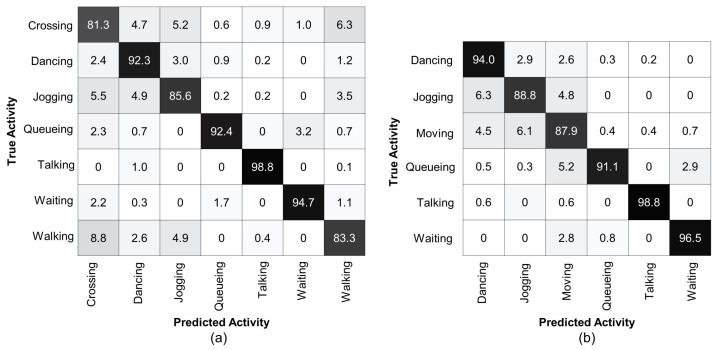
Confusion matrix for group activity recognition using augmented Collective Activity Dataset. (**a**) denotes the confusion matrix for the original seven classes. Most confusion occurs in distinguishing between the Crossing and Walking classes. (**b**) denotes confusion matrix after merging the Crossing and Walking classes into a single Moving class. Our method achieves over 87% accuracy for each group activity class.

For further analysis of our method, we used an augmented dataset of the Collective Activity Dataset with two additional outdoor activity classes such as Dancing and Jogging , see Figure 5a. It can be seen that the Crossing class achieves the lowest accuracy of 81.3% and is highly confused with the Jogging and Walking classes with a ratio of 5.2% and 6.3% , respectively. This is because of the similar physical appearance of human bodies in these classes. Similarly, the Jogging class is confused with the Crossing and Walking classes with a ratio of 5.5% and 3.5% , respectively. In addition, the Walking class is confused with the Crossing and Jogging classes with a ratio of 8.8% and 4.9% , respectively. We again merged the Crossing and Walking classes into the Moving class and implemented our method on the merged dataset, see Figure 5b. It is clear that the proposed method attains improved accuracies for all classes of the merged dataset, except for Queueing class, which slightly deteriorated from 92.4% to 91.1% .

**Figure 6:**
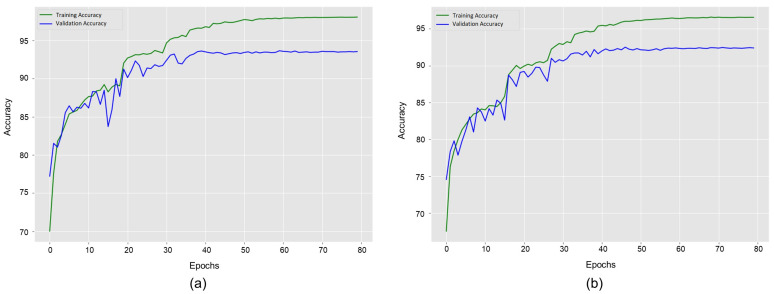
Model accuracy curves of group activity recognition using the training and validation datasets on the Collective Activity Dataset. (**a**) denotes the learning curves for the original dataset with the five classes. (**b**) denotes the learning curves for the augmented dataset with the six merged classes.

Figure 6 illustrates the accuracy convergence of our method concerning epochs using different dataset settings of the Collective Activity Dataset. Figure 6a shows results for five classes dataset such as Crossing , Queueing , Taking , Waiting , and Walking . It shows the highest validation accuracy of 93.6% at the 60th epoch. Training accuracy starts at 70.0% and goes up to 98.0% . Then, training accuracy convergence becomes stable. Validation accuracy starts at 77.2% and goes up to 93.6% . It slightly went down at various epochs during the first 40 epochs and then became almost stable. Meanwhile, training loss for this dataset starts from 0.73 and decreases up to 0.06 with increasing epochs. Figure 6b shows results for the six classes dataset such as Dancing , Jogging , Moving , Queueing , Taking , and Waiting . It depicts the highest validation accuracy of 92.5% at the 72nd epoch. Training accuracy starts at 67.5% and goes up to 96.6% . Validation accuracy starts at 74.6% and goes up to 92.4% . It slightly went down at various epochs during the first 50 epochs and then became nearly stable. The value of training loss for this dataset reduces from 0.85 to 0.10 as the epochs increase.

### 4.5. Limitations and Future Work

The proposed 3DMesh-GAR method is the first to implement real-time multi-person activity recognition using 3D body mesh. Nonetheless, it has some limitations, which will be addressed in this subsection.

**Figure 7:**
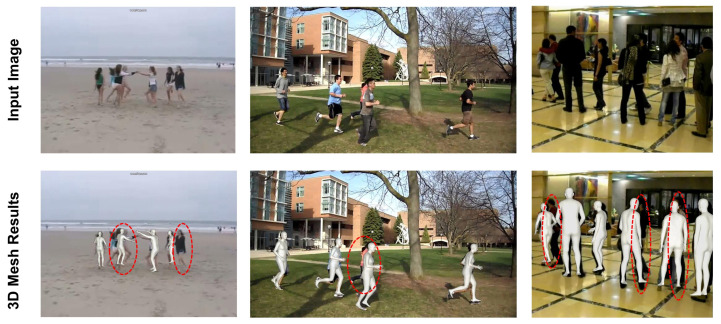
Qualitative analysis of 3D Mesh reconstruction in highly occluded and long distance scenarios.

As our 3D body mesh reconstruction approach can process multi-persons simultaneously without relying on bounding boxes for level human detection, it is naturally sensitive to person scale variations, which limits its applicability on the wild images. Moreover, our mesh reconstruction is based on 2D human representation, such as 2D Body Center Heatmap, which makes it difficult to learn the mapping function. Our method successfully reconstructs the 3D human body meshes even in the presence of person-to-person occlusion. However, it still shows the limitation of creating meshes in extremely occluded scenarios and in the presence of long-distance between persons and camera, as shown in Figure 7. All persons with missing 3D body meshes are highlighted with red circles.

Because our deep network for action recognition in Stage III is trained with mesh features, our major focus in future studies will be to improve the mesh reconstruction network in Stage I. To do so, we can replace the 2D body center heatmap with 3D skeletons for multi-person detection in the frame. Thus, the efficiency of the mesh reconstruction network can be improved by collaborating the 3D joints and 3D body meshes. In addition, we will extend our work for short abnormal actions detection in a video sequence of long normal actions.

## 5. Conclusions

Autonomous group activity recognition has become a growing research field in the past few years and has achieved impressive progress. Activity recognition methods based on deep learning approaches are playing a vital role in GAR. Thus, this paper proposed 3DMesh-GAR, an efficient and flexible 3D human body mesh-based method for group activity recognition. It takes RGB image frames as input and product 3D body meshes using their body center heatmaps and mesh parameter maps. The 3D mesh features are very simple and easy to train with a linear neural network. A deep artificial neural network architecture has been developed and optimized to learn and recognize group activities from the proposed description in an end-to-end manner. We evaluate the proposed method on a benchmark group activity recognition dataset and establish a new state-of-the-art performance. Various experiments and results analysis show how an intuitive and simple 3D mesh-based method regarding information representation can be successful for high-level feature extraction from a complex dataset.

## Figures and Tables

**Table 1:** Collective activity dataset comparison with state-of-the-art methods for group activity recognition. **Bold** denotes the best results.

Methods	Backbone	Group Activity
Lan et al. [33]	None	79.7%
Choi and Savarese [11]	None	80.4%
Deng et al. [12]	AlexNet	81.2%
Ibrahim et al. [13]	AlexNet	81.5%
Hajimirsadeghi et al. [32]	None	83.4%
Azar et al. [41]	I3D	85.8%
Li and Chuah [16]	Inception-v3	86.1%
Shu et al. [35]	VGG16	87.2%
Qi et al. [55]	VGG16	89.1%
Ehsanpour et al. [107]	I3D	89.4%
Wu et al. [42]	Inception-v3	91.0%
Gavrilyuk et al. [65]	I3D	92.8%
Ours (3DMesh-GAR)	ResNet-50	**93.6%**

**Table 2:** Comparison of each activity class recognition of the CAD dataset with the previous state-of-the-art method. **Bold** denotes the best results (%).

Methods	Crossing	Queueing	Talking	Waiting	Walking
Gavrilyuk et al. [65]	83.3	**100**	**100**	96.1	**88.1**
Ours	**88.8**	95.2	99.7	**96.8**	86.7

**Table 3:** Comparison of five different activity classes recognition of the NTU60 dataset with the previous state-of-the-art method. **Bold** denotes the best results (%).

Methods	Back-Pain	Bow	Brush-Teeth	Cheer-Up	Shaking-Hands	Overall Accuracy
Huang et al. [108]	88.7	90.9	85.9	87.7	**93.8**	89.4
Ours	**90.2**	**93.7**	**88.1**	**95.0**	91.9	**92.2**

## Data Availability

The data presented in this study are available on request from the corresponding author.

## Abbreviations

The following abbreviations are used in this manuscript:

GAR	Group Activity Recognition
3DMesh-GAR	3D Mesh-based Group Activity Recognition
GCNs	Graph Convolutional Networks
RNNs	Recurrent Neural Networks
CNNs	Convolutional Neural Networks
DNNs	Deep Neural Networks
D-ANN	Deep Artificial Neural Network
GNNs	Graph Neural Networks
LSTM	Long Short-Term Memory
NLP	Natural Language Processing
SVMs	Support Vectors Machines
ARG	Actor Relation Graph
SMPL	Skinned Multi-Person Linear model
R-CNN	Region-based Convolutional Neural Networks
3DMPPE	3D Multi-Person Pose Estimation
LCR-Net++	Localization Classification Regression Network
LoCO	Learning on Compressed Output
GPUs	Graphics Processing Units
ReLU	Rectified Linear Units
